# Rheological Investigation of Welding Waste-Derived Graphene Oxide in Water-Based Drilling Fluids

**DOI:** 10.3390/ma15228266

**Published:** 2022-11-21

**Authors:** Rabia Ikram, Badrul Mohamed Jan, Waqas Ahmad, Akhmal Sidek, Mudasar Khan, George Kenanakis

**Affiliations:** 1 Department of Chemical Engineering, Faculty of Engineering, University of Malaya, Kuala Lumpur 50603, Malaysia; 2 Institute of Chemical Sciences, University of Peshawar, Peshawar 25120, Khyber Pukhtunkhwa, Pakistan; 3 Petroleum Engineering Department, School of Chemical and Energy Engineering, Faculty of Engineering, Universiti Teknologi Malaysia, Johor Bahru 81310, Malaysia; 4 Institute of Electronic Structure and Laser, Foundation for Research and Technology-Hellas, N. Plastira 100, Vasilika Vouton, GR-700 13 Heraklion, Crete, Greece

**Keywords:** industrial waste, nanomaterials, drilling fluids, graphene oxide, rheology, filtrate loss

## Abstract

Throughout the world, the construction industry produces significant amounts of by-products and hazardous waste materials. The steel-making industry generates welding waste and dusts that are toxic to the environment and pose many economic challenges. Water-based drilling fluids (WBDF) are able to remove the drill cuttings in a wellbore and maintain the stability of the wellbore to prevent formation damage. To the best of our knowledge, this is the first study that reports the application of welding waste and its derived graphene oxide (GO) as a fluid-loss additive in drilling fluids. In this research, GO was successfully synthesized from welding waste through chemical exfoliation. The examination was confirmed using XRD, FTIR, FESEM and EDX analyses. The synthesized welding waste-derived GO in WBDF is competent in improving rheological properties by increasing plastic viscosity (PV), yield point (YP) and gel strength (GS), while reducing filtrate loss (FL) and mud cake thickness (MCT). This study shows the effect of additives such as welding waste, welding waste-derived GO and commercial GO, and their amount, on the rheological properties of WBDF. Concentrations of these additives were used at 0.01 ppb, 0.1 ppb and 0.5 ppb. Based on the experiment results, raw welding waste and welding waste-derived GO showed better performance compared with commercial GO. Among filtration properties, FL and MCT were reduced by 33.3% and 39.7% with the addition of 0.5 ppb of raw welding-waste additive, while for 0.5 ppb of welding waste-derived GO additive, FL and MCT were reduced by 26.7% and 20.9%, respectively. By recycling industrial welding waste, this research conveys state-of-the-art and low-cost drilling fluids that aid in waste management, and reduce the adverse environmental and commercial ramifications of toxic wastes.

## 1. Introduction

The construction and steel industries produce significant amounts of by-products and welding wastes around the planet [1]. Welding waste is produced during the welding process in the form of vitreous material or slag, of red or black color, which is a non-biodegradable, toxic and hazardous waste material [2,3]. These wastes can be recycled and reused as a constructive material by the proper management of welding waste [4]. For instance, welding wastes can be reused by extracting the valuable minerals inside them through physical or chemical mineral-processing techniques [5]. In addition, welding wastes can be utilized in land filling and in the production of cement [6]. On construction sites, welding waste can be added to road surfaces or the pavements of airports as an asphalt mixture additive [7]. Thus, recycling welding wastes not only conserves the mineral resources but also protects the environment [8].

Steel wastes have similar properties to bentonite clay. Bentonite clay is widely used in the petroleum industry. Almost 80% of drilling fluids consist of bentonite clay. Drilling fluids perform many functions in the drilling operation, such as transporting the drill cuttings from the bottom of a wellbore to the surface; suspending cuttings; maintaining the wellbore pressure of the formations to prevent blow out; cooling and lubricating the drill bit and sealing permeable formations [9]. High amounts of aluminum and titanium dioxide (TiO_2_) are found in welding waste and can improve the rheological properties of WBDF [10]. Such waste contains similar ingredients to commercial fluid-loss additives, for example, TiO_2_. The addition of TiO_2_ to drilling fluids increases viscosity, while reducing FL and MCT. Due to its unique properties, TiO_2_ enhances thermal conductivity and acts as a viscosifier as compared to conventional drilling fluids [11].

Previous data shows that total steel demand grew to hundreds of millions of metric tons worldwide [12]. In metallurgical processes, different types of slags are generated as by-products or large amounts of residues in metal incineration processes [13]. A noteworthy analysis was performed to investigate solid waste composition and generation rates in six vocational college welding workshops in Malaysia [14]. The data revealed that welding waste was composed of scrap metal, metal dust, welding electrodes and grinding disks which constituted 92.89, 3.64, 3.07 and 0.4 percent of the total welding waste, respectively. The total welding waste generation rates varied from 59.57 to 117.63 kgw^−1^ across the study workshops, with an average of 83.42 kgw^−1^. Per capita generation rates varied from 0.60 to 1.90 kgw^−1^, with an average of 1.23 kgw^−1^. These data showed the potential and environmental effects of welding waste due to the presence of hazardous constituents which were known to contain a variety of metals and metal oxides [14,15].

In recent years, the usage of GO as a drilling mud additive was established. Due to its high surface area, stability, water resistance and strong mud formation properties, it possesses the super-efficient capability of preventing the leakage of drilling mud into the wellbore [16,17]. A variety of studies presented the addition of GO as an emerging additive for WBDF to enhance rheological properties, such as FL and MCT. Nevertheless, none of the previous studies evaluated the effects of welding waste, or its derivatives as additives, in drilling fluids [18,19]. By choosing the optimal concentration of GO, the properties and hydraulics of drilling fluids can be enhanced [20]. GO helps create an ideal drilling mud as it can prevent the invasion of drilling mud into the formation. Thus, GO-based drilling fluid can significantly reduce friction between the borehole and the drill pipe due to its fine film-forming capabilities [21].

Drilling fluid serves as the material to cool the drill bit during a long drilling operation. When there is contact between the drilling fluids and formation, minimal impact on the mechanical properties of the formation itself is essential. [22]. In order to complete a drilling operation successfully, maintaining an open hole is very important. The addition of welding waste, and its derived GO as additives, allows the drilling fluid to serve as a hole cleaner to eliminate the cuttings from the bottom of the well hole and to control subsurface pressure [23]. The usage of bentonite and different expensive additives used in drilling fluid formulations is expected to decrease as these are replaced with sustainable waste-derived additives. Moreover, this will reduce the residual effects of welding and construction waste, thereby protecting the environment [24]. FL is commonly known as the loss of the mud filtrate into the porous permeable formation. The invasion of drilling fluid is due to the presence of a higher hydrostatic pressure in the wellbore than the pressure in the formation. This is a critical issue during a drilling operation as it leads to many problems, such as formation damage and pipe sticking. If mud filtrate invades the formation, production of the hydrocarbon decreases as the permeability, capillary pressure and wettability are affected. Thus, non-toxic, low cost and environmentally safe additives are added to the drilling fluids to reduce fluid loss into the formation [25].

The application of waste materials in the petroleum industry is important to reduce the negative impact on society. Conventional drilling fluids produce thick mud cake and a significant amount of filtrate loss (FL) which eventually damage the reservoir [26]. Furthermore, current industry practice uses commercial GO which is expensive at around MYR 1000 per few grams, which increases the investment cost of drilling operations. The availability of carbonaceous industrial waste also triggers the initiative to turn such industrial waste into value-added products [27]. To the best of our knowledge, this is the first study using welding waste and welding-waste-derived GO in WBDF. The aim of the research is to recognize the usage of welding waste to improve its filtration properties as a fluid loss agent. This work, therefore, endeavors to fulfill the research gap by proposing the recycling of welding waste and its derived GO to investigate the effect of drilling fluids, compared to basic water bentonite suspensions. The performance of rheological and fluid loss properties was conducted by incorporating welding waste and welding waste-derived GO and comparing them to commercial GO prepared at three different levels (low/medium/high 0.01, 0.1 and 0.5 wt%). Firstly, the unwanted industrial welding waste was converted into GO using a modified Hummers method. Characterization of the fabricated GO was then performed using XRD, FTIR, FESEM and EDX analysis. Furthermore, these additives successfully demonstrated the effectiveness of waste additives following examination of their rheological properties under American Petroleum Institute (API) standards; the properties included plastic viscosity (PV), yield point (YP), gel strength (GS), FL and mud cake thickness (MCT). Finally, we also determine the optimized amount of welding waste and welding waste-derived GO additives that produced the lowest FL and thinnest mud cake.

## 2. Materials and Methods

### 2.1. Chemicals and Reagents

All the chemicals used were of analytical grade and used as received. Sulfuric Acid (H_2_SO_4_ 98%), Hydrochloric Acid (HCl 37%) and Sodium Nitrate (NaNO_3_) were purchased from Riedel-de-Haen. Potassium Permanganate (KMnO_4_) was supplied by Merck, and Potassium Hydroxide (KOH) and Hydrogen peroxide solution 35% (H_2_O_2_) from Sigma-Aldrich. Deionized water was used throughout the experiments.

The following components of drilling fluids were used: Sodium hydroxide (purity ≥ 99 wt%), Potassium chloride (purity ≥ 99 wt%) and Carboxymethylcellulose (purity ≥ 99 wt%) were acquired from Sigma-Aldrich, St. Louis, MO, USA. Bentonite and barite (purity 91–93 wt%) were provided by M-I SWACO, Malaysia. Distilled water was used to prepare all aqueous solutions with no further purification.

### 2.2. Synthesis of Welding Waste-Derived GO

A welding-waste sample was collected from a local welding workshop. The waste sample was a gray colored powder material with a high density due to the presence of metallic residues. The welding-waste sample was first carbonized in a muffle furnace, in the absence of oxygen, at 300 °C for 3 h. The carbonized char was ground to a fine powder and screened through a 125 µm mesh size sieve. The carbonized mass was then pyrolyzed under continuous flow of N_2_ in a stainless-steel tubular reactor. About 5 g of sample was placed in the reactor tube which was connected to the N_2_ supply. The tube was inserted into a tubular furnace and heated at 550 °C for 1 h. The sample was allowed to cool within the reactor and then stored in vials [28].

To remove the metallic, mineral and ash residue, the sample was first treated with KOH solution (in a ratio of 1:1.4 *w*/*w*) under vigorous treatment for 4 h in a beaker. The suspension was allowed to settle for a few hours and the clear aqueous layer was removed. The sample was then suspended in distilled water under vigorous stirring; the suspension layer was quickly decanted and the bottom residues were discarded. The suspension was allowed to settle and then filtered to allow the residue to be collected. The residue was excessively washed with 0.1 N HCl solution and distilled water until the pH of washing was neutralized. The pyrolyzed carbon residue was dried in an oven at 60 °C for 5 h and then stored in vials.

**Figure 1:**
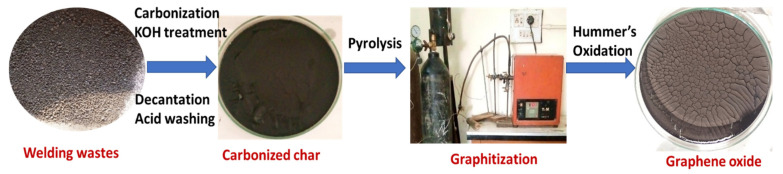
Stepwise synthesis of GO from welding wastes.

GO was prepared from pyrolyzed carbon powder via an improved Hummers’ method [16,29,30]. About 5 g of pyrolyzed carbon derived from welding waste was added to a flask containing a solution of concentrated H_2_SO_4_ (92 mL) and NaNO_3_ (4 g), placed in an ice bath and stirred for 10 min. About 15 g of KMnO_4_ was slowly added to the suspension under continuous stirring; the temperature rose due to the exothermic nature of the reaction, and was maintained at less than 30 °C in the ice bath. After this, 2–3 mL of H_2_O_2_ was dropwise added to the suspension and the temperature rapidly rose but was controlled to about 90 °C. The color of the slurry turned brown; the flask was covered and allowed to stand overnight. About 180 mL of distilled water was added to the mixture and slowly heated to 90–95 °C for 30 min; the brown-colored residue (GO) was collected through filtration, excessively washed with distilled water and then dried in oven at 60 °C for 5 h [31]. The sequences of steps involved in the synthesis of GO are shown in Figure 1 below.

### 2.3. Characterization of GO

The welding waste-derived GO was characterized by FTIR, FESEM, EDX and XRD analysis. The composition and crystallinity of GO was investigated through X-ray diffractometer (XRD; model JDX-9C, JOEL, Tokyo, Japan) using CuKα radiation (1.54178 A° wavelength) and an Ni filter. The surface morphology and elemental composition was studied by FESEM and EDX analysis through Scanning Electron Microscope (Model JEOL-Jsm-5910; Tokyo, Japan). The functional group composition was evaluated by FTIR spectrophotometer (Schimadzu-A60, Tokyo, Japan).

### 2.4. Drilling Fluid Preparation

Three types of WBDF were prepared in this research; these were basic WBDF with (1) commercial GO, (2) raw welding waste and (3) welding waste-derived GO as the additives. The different additives were added to the base fluid using different concentrations—0.01, 0.1 and 0.5 ppb—as described in previous research work [32]. During the formulation of the base fluid, water was added to the bentonite to create a hydrated slurry, followed by the addition of the remaining components. Table 1 shows the formulation of the WBDF including each material, required mixing time and mixing order for all additives.

### 2.5. Evaluation of Rheological and Filtration Properties

A Fann Model 35 Viscometer (Houston, TX, USA) and an Anton Paar rheometer (Germany) were operated at room temperature to measure the rheological properties. The viscometer was used to measure the PV, YP and GS of the drilling fluid at rotor speeds of 300 rpm and 600 rpm. The dial readings for both speeds are recorded as ∅_300_ and ∅_600_. For the GS, the drilling fluid was stirred at 600 rpm until it reached a steady dial reading value. The drilling fluid sample was then held for 10 s and the motor was stopped. The maximum reading was achieved and recorded as 10 s of GS; the same steps were repeated for 10 min of GS.

The filtration test was piloted by pouring the mud sample into the cell to within 1/2 inch of the top, and the filtrate was collected using a dry graduated cylinder placed under the drain tube. An OFITE filter press was used for this test. The system used N_2_ to supply pressure and a standard filter paper. The pressure relief valve was opened and began to record the FL as a function of time. According to the API standard, the operating pressure was 100 psi and the temperature was atmospheric (77 °F). After 30 min, the FL was measured in cubic centimeters (to 0.1 ccs). The MCT was measured using a digital Vernier caliper, model Mitutoyo 500-197-20, to the nearest 1/32 inch [32,33].

## 3. Results and Discussion

### 3.1. Characterization of GO

**Figure 2:**
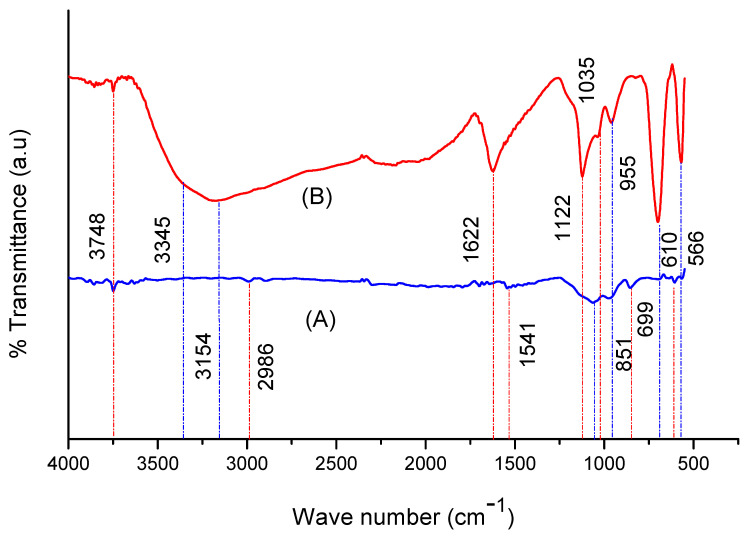
FTIR spectra of (**A**) welding wastes and (**B**) GO.

The FTIR spectra of welding wastes and of GO derived from welding wastes are shown in Figure 2. The spectrum of welding wastes shows a weak absorption band at 3748 cm^−1^ corresponding to the O-H bond of Si-O-H. Bands appeared at 2986 cm^−1^, showing the C-H bond, and at 1700, showing C=O; multiple bands appeared in the range of 1660 cm^−1^–1541 cm^−1^, corresponding to aromatic C=O configurations. Peaks at 1058 cm^−1^ and 955 cm^−1^ show an Si-O stretching vibration and Si-O-Al vibrations, whereas the bands appearing between 800 and 500 show metals-oxygen bonds [4,34]. These results show that the welding waste consists of silicates, aluminates and oxides of various metals, along with some proportion of graphite.

The FTIR spectrum of GO exhibits prominent bands positioned at 3748, 3345 and 3154 cm^−1^, which correspond to the O-H stretching vibrations of Si-OH, O-H carboxylic acids and C-H aromatics [5,31]. The absorption bands positioned at 1622, 1122, 1035 and 955 cm^−1^ show C=C aromatics, carboxyl O=C-O, Si-O and Si-O-Al, respectively [5,35], whereas the FT-IR absorption bands appearing in the range of 610–500 cm^−1^, correspond to various metal-oxygen linkages [6,7,36,37]. The results show that the GO sample also contains impurities such silica, alumina and several metal oxides.

**Figure 3:**
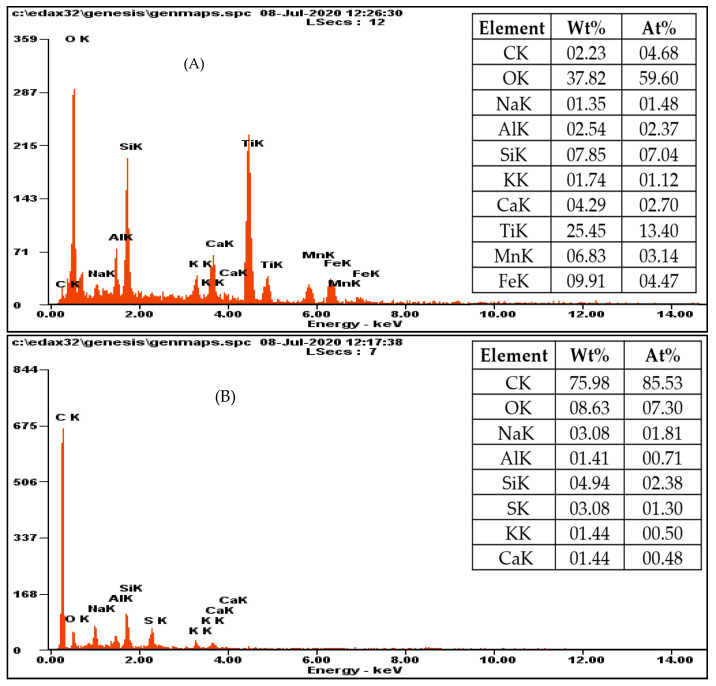
EDX profiles of (**A**) welding wastes and (**B**) GO.

The elemental composition of the welding-waste sample and GO prepared from welding waste was determined through EDX analysis. The EDX spectra of the samples and the % weight and atomic % values of various elements in the samples are displayed in Figure 3. These results reveal that the welding-waste sample contains various elements as their oxides, including Ti, Mn, Fe, Si, Ca, K, Na and Al, and of which Ti, Si, Fe and Mn are present as a high atomic % i.e., 13, 7, 4 and 3%, respectively. About 4 atomic % carbon in the sample is also present in the sample, the form of graphite. The welding electrode generally consists of a metal rod made of steel or wrought iron; the flux material around it contains cellulose, silica and oxides of various metals, such as Fe, Mn, Al, Ti, Ca and others [8,38]. During arc welding, the electrode transforms into residues of oxides. It was shown that the welding flux changes into granular powder during arc welding, and consists of alumina, silica, and oxides of Ca, Mn, Fe, Ti and other minerals [9,39]. The composition of welding wastes agrees with the composition of the components of the electrode. The elemental composition of the GO derived from welding wastes includes about 85% carbon and 7% oxygen, which confirms the synthesis of GO. It is clear from the results that Ti, Fe and Mn are not present in the sample where the other metals are present in very smaller quantities. It is inferred that during the synthesis of GO, the carbon content was enriched, and the other metals leached from the welding residue through acid treatment and the decantation processes. The presence of S in the GO sample is also an indication of contamination from the sulfuric treatment during the synthesis process.

**Figure 4:**
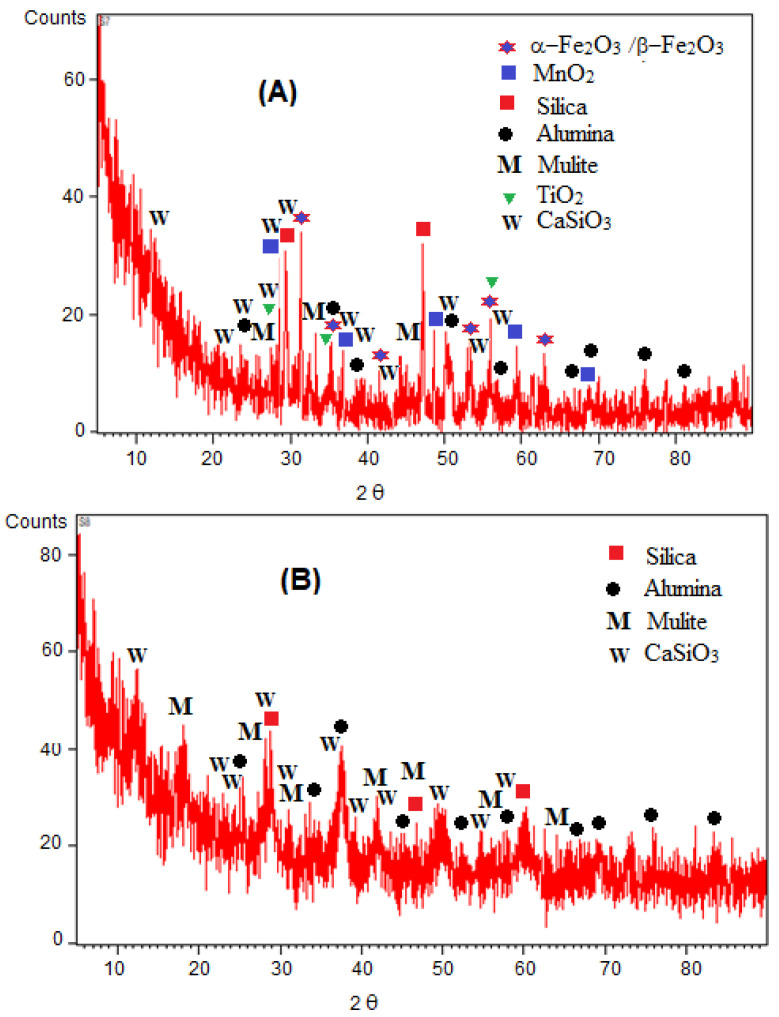
XRD patterns of (**A**) welding wastes and (**B**) GO samples.

The XRD pattern of the welding wastes and GO is given in Figure 4. The XRD pattern of welding wastes shows an amorphous hump and some sharp and intense peaks; the amorphous pattern appears at 2θ of 0 to 20° which indicates the presence of powder graphite. The high-intensity prominent peaks represent the presence of Fe_2_O_3_, MnO_2_, TiO_2_, silica and alumina as major components. The other less intense peaks indicate the presence of mullite and wollastonite in smaller proportions. The components of welding wastes exhibited by the XRD analysis agree with the literature reports [9,10,39,40].

In the case of GO, the XRD pattern shows a typical amorphous configuration of GO with a sharp peak at 10.5° 2θ [5,35]. Other configurations indicated by the XRD patterns of GO include silica, alumina, mullite and calcium silicate. As per EDX analysis, other minerals containing Na, S and K may also be present in the welding waste-derived GO. However, due to their small concentrations, the corresponding peaks do not appear in the XRD patterns.

**Figure 5:**
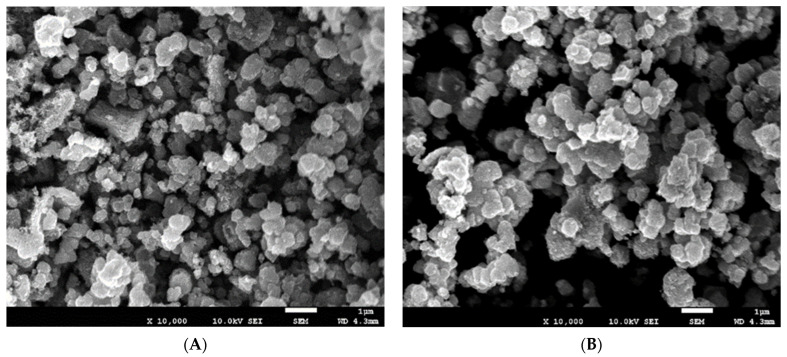
FESEM micrographs of (**A**) Welding waste sample and (**B**) GO.

The FESEM micrographs of welding wastes and GO derived from welding wastes are displayed in Figure 5 which illustrates that both samples exhibit an identical granular morphology. However, the granular size of the welding waste sample is non uniform, wherein the particles of different sizes ranging from nanosized grains to large lumps of two microns can be seen. Also, the grains seem agglomerated or glued together, which may be attributed to the presence of oxides of various metals. However, the GO sample represents regular-shaped, uniformed-sized particles with an average grain size of about half a micron.

### 3.2. Properties of Drilling Fluids

The rheological properties of drilling fluids such as PV, YP, 10 s and 10 min GS, API FL and MCT were determined as summarized in Table 2, Table 3 and Table 4 below.

The rheological properties of WBDF with commercial GO as the additive are tabulated in Table 2.

The rheological properties of WBDF with raw welding waste as the additive are tabulated in Table 3.

The rheological properties of WBDF with welding waste-derived GO as the additive are tabulated in Table 4.

#### 3.2.1. Plastic Viscosity

**Figure 6:**
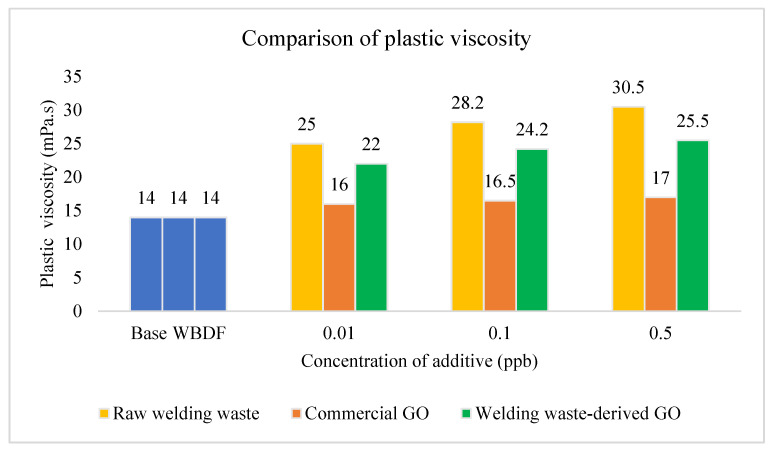
PV comparison of WBDF using different additives.

Figure 6 represents the comparison of plastic viscosity of raw welding waste, welding waste-derived GO and commercial GO-based drilling fluids with different concentrations. The results show that the PV of 0.5 ppb raw welding waste drilling fluid is 30.5 cP, which is the highest among them all. Drilling fluids with the presence of welding wastes increase dramatically compared with the base drilling mud and commercial GO drilling fluid. This might be due to the presence of the solid form welding waste, which increases the flow resistance of the drilling fluids [11,41]. PV increases when the mechanical friction between solids and liquids increases.

#### 3.2.2. Yield Point

**Figure 7:**
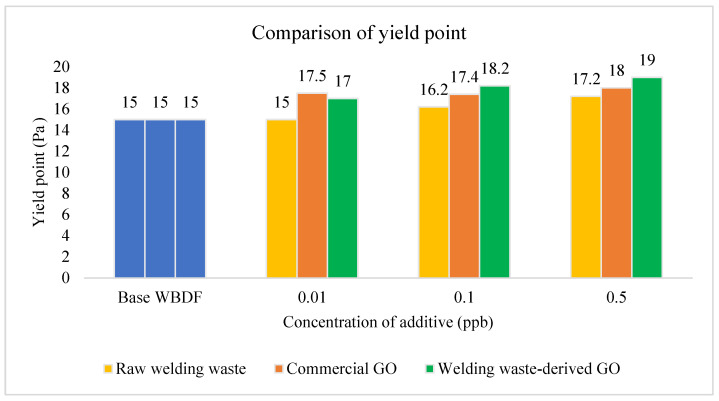
YP comparison of WBDF using different additives.

A comparison of YP for the different drilling fluid formulations at different concentrations is presented in Figure 7. The trend for all types of drilling fluids are that the YP increases with increasing concentration, except for 0.1 ppb of commercial GO. The lifting capacity of a high YP is better but would increase the cost of the power when the drilling fluid is pumped in the wellbore. Drilling fluids with high YP indicate that it has a higher suspension. The rock cuttings would not sink to the bottom when the drilling operation is stopped. Stuck pipes and lost circulation can be prevented if the YP of the drilling fluids are controlled. The YP of welding waste-derived GO drilling fluid increased from 15 $\mathrm{Ib}/100{\;\mathrm{ft}}^{2}$ to 19 $\mathrm{Ib}/100{\;\mathrm{ft}}^{2}$ when its concentration was 0.5 ppb. An increasing YP can improve the dynamic cutting suspension and efficiency of the hole cleaning of the drilling fluids [12,42].

#### 3.2.3. Gel Strength

**Figure 8:**
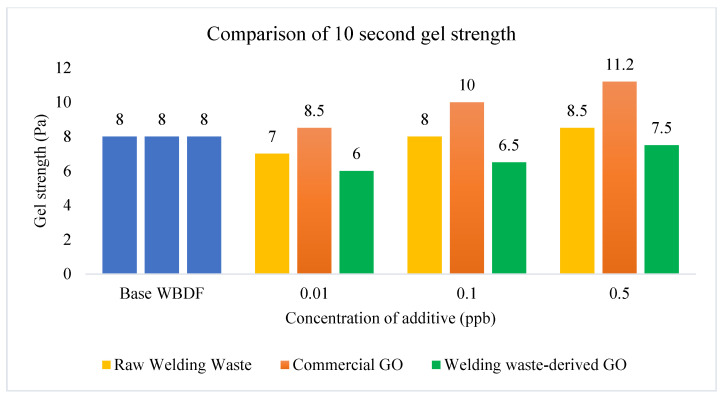
10 s GS comparison of WBDF using different additives.

**Figure 9:**
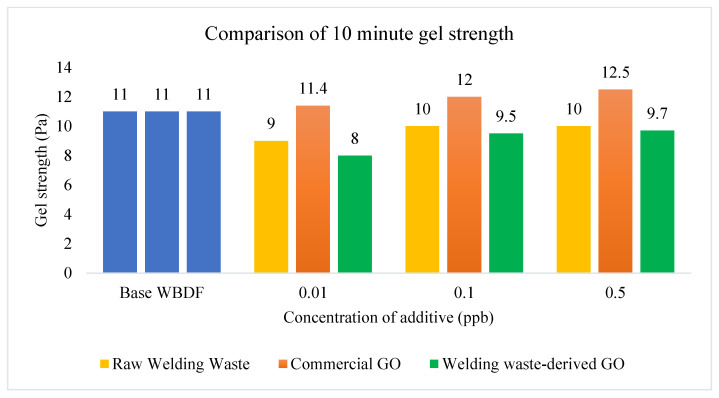
10 min GS comparison of WBDF using different additives.

GS is related to the viscosity of the drilling fluid and affects the ability of drilling fluids to lift rock cuttings [43]. Figure 8 and Figure 9 show that GS increased with the increasing concentration of additives when different types of additives were added into WBDF. The 10 s GS and 10 min GS of commercial GO based drilling fluid increased to 11.2 $\mathrm{Ib}/100{\;\mathrm{ft}}^{2}$ and 12.5 $\mathrm{Ib}/100{\;\mathrm{ft}}^{2}$, respectively, when the concentration of additives was 0.5 ppb. Drilling fluids have higher suspension power with a higher GS when the drilling operation is stopped. For the 10 s GS in Figure 8, the GS deceased slightly when raw welding waste and welding waste-derived GO were added into WBDF. From Figure 9, it can be seen that the 10 min GS increased gradually as the concentration of additives increased.

#### 3.2.4. Filtrate Loss

**Figure 10:**
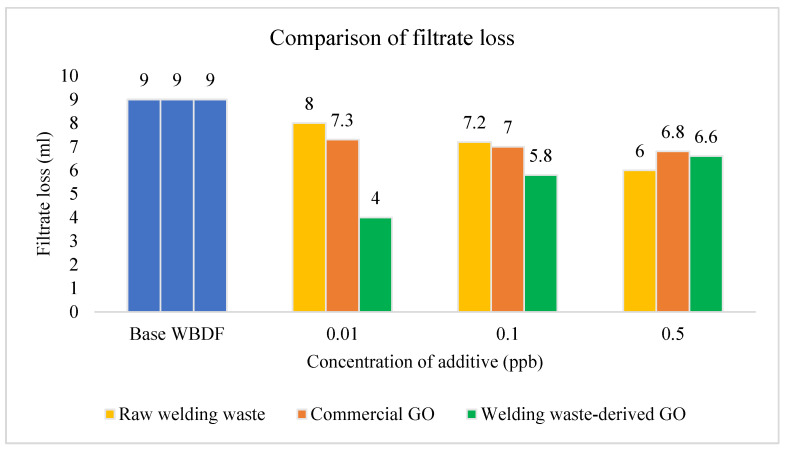
FL comparison of WBDF using different additives.

The FL of the three types of drilling fluid at the different concentrations of 0.01 ppb, 0.1 ppb and 0.5 ppb is shown in Figure 10. The base fluid displayed a filtrate loss volume of 9 mL. The fluid loss was significantly reduced to 6.6, 5.8 and 4 mL after the addition of 0.5, 0.1 and 0.01 wt% of GO nanoparticles to the WBDF, respectively, as shown in Figure 9. Raw welding waste-derived GO drilling fluid with 0.01 ppb shows the best result as the FL decreased from 9 mL to 4 mL—the highest reduction. The GO nanoparticles remarkably minimize the permeability and porosity of the mud cake [44]. By consolidating the attractive force between the particles, the volume of FL is reduced. On the other hand, when the amount of welding waste-derived GO was increased, the FL also increased.

The usage of GO as a drilling fluid additive is likely because it has a special surface area of 2391 m^2^/g [13]. This acts as a solid penetrating layer that can make the formation of mud cake in drilling mud stronger, thereby preventing the mud from flooding into the formation. Furthermore, GO possesses another interesting characteristic, namely separation of the solution and the formation of a strong paper-shaped material capable of efficiently preventing drilling mud leaks into the well wall. For instance, dispersed GO flakes can be filtered out of the solution and pressed to make a strong paper-like material, which results from the robust tile-like interlocking of the flakes. This could be beneficial for making a thin impermeable film to prevent FL in the wellbore [23,45].

It is believed that FL is affected by the concentration of nanomaterials added after a specific point as 0.01 ppb is the optimum concentration of waste-derived GO drilling fluid with the lowest FL. Excessive nanomaterials added to drilling fluid may reduce the effectiveness, make it costlier and increase the chance of formation damage [46,47].

#### 3.2.5. Mud Cake Thickness

**Figure 11:**
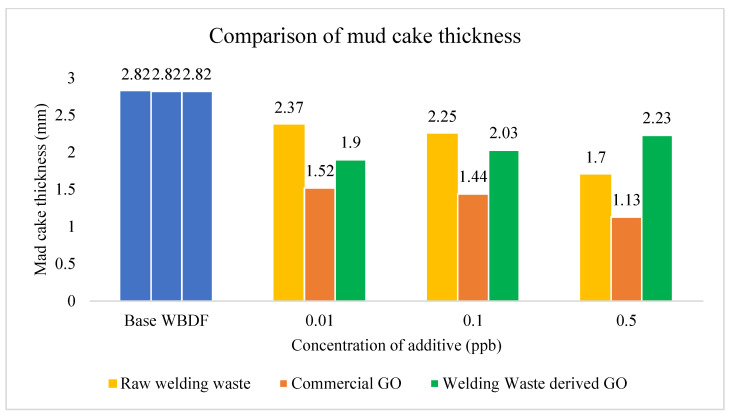
MCT comparison of WBDF using different additives.

The optimum thickness of mud cake helps to increase the stability of the wellbore and decrease mud invasion; hence, thin mud cake is recommended [48]. This is due to thick filter cake increasing the chance of the drill pipe becoming stuck when in contact with the mud cake under pressure. Figure 11 shows the thickness of mud cake with the addition of different amounts of raw welding waste, commercial GO and welding waste-derived GO. The effectiveness of the drilling fluid is closely related to the permeability and porosity of the mud cake. As shown in Figure 11, a decrease in fluid loss volume results in a decrease in MCT. It is clear that by adding the lowest GO concentration (0.01 wt%) to WBDF, the lowest FL volume was established, and the thinnest mud cake was obtained, demonstrating significant enhancement by lowering the FL by 55.6% when compared with basic WBDF. Similarly, MCT was reduced to 32.6% using welding waste-derived GO. As a result, GO nanoparticles were critical in blocking the nanopores in the filter cake made of bentonite particles. For commercial GO and raw welding waste-derived GO, significant results are obtained, as MCT is reduced with a minimum amount of additive. However, large graphene-cut stacks in the water medium experience issues with WBDF. Therefore, GO, which is more water resistant and possesses the same layered morphology, is capable of forming the desired mud cake. Since GO sheets are well exfoliated, they could be added at substantially lower concentrations than clay-based additives to obtain the desired performance. More prominently, the nanometer thickness of the GO flakes could also result in much thinner filter cakes than those obtained using clay-based materials. The thickness of a filter cake is directly correlated with the differential torque needed to rotate the pipe during drilling operations, to the drilling time and to drilling costs. In this work, GO is further appealing as it offers the prospect of a waste-derived mechanism and inexpensive technology [20,22,45].

Both additives were able to form the thinner mud cake compared with the conventional WBDF. From this study, it can be concluded that waste-derived GO can prominently improve the filtration properties of WBDF [49,50].

## 4. Conclusions

In this work, unwanted welding waste-derived GO was prepared in a novel way using a modified Hummers’ method to improve the filtration properties of WBDF. A comparison of three additives including raw welding waste, welding waste-derived GO and commercial GO was conducted, using three different concentrations i.e., low (0.01 wt%), medium (0.1 wt%) and high (0.5 wt%) under API standard conditions. The following conclusions can be drawn based on this research;

- The effect of the lowest concentration of welding waste-derived GO showed the lowest FL up to 55.6%.
- The remarkable reduction of MCT was obtained by adding highest concentration of raw welding waste (39.7%). However, the highest concentration of welding waste-derived GO and commercial GO showed reductions of 20.9% and 59.9%, respectively.
- The lowest concentration of commercial GO showed an MCT reduction of 46.1% compared with welding waste-derived GO, which showed a reduction of up to 32.6%. The results verify that the use of novel nanocomposites in drilling fluids is capable of increasing efficiency, while reducing operating cost. These waste-derived economical additives can be excellent alternatives to commercial GO.
- The linked structure of GO allows more water to be trapped between layers. This causes an increase in viscosity and yield stress, while reducing fluid filtration.
- The addition of waste-derived GO could be a useful substitute of commercial GO.
- The major cost to produce GO is the cost of chemicals, equipment and labor. The chemicals required include H_2_SO_4_, KMnO_4_, NaNO_3_ and H_2_O_2,_ which are all abundantly available in the open market at very low prices. Moreover, these are commonly used chemicals and are available in almost every lab. The cost of these consumables is same as for commercial GO and GO derived from welding wastes. These only differ in the supply of raw materials. For commercial GO, the raw material is pure graphite, which must be imported at high cost, whereas for synthesis of GO from welding waste, the raw material is a waste product and available free of cost. Thus, the total production cost of welding waste-derived GO is much lower than that of t commercial GO.
- This research focused on the conversion of hazardous waste into GO which validates the conversion of many other waste materials into graphene derivatives and potential filtrate loss agents. Thus, this study paves the way for utilizing a variety of waste sources in sustainable and cost-effective drilling operations. The availability of carbonaceous industrial waste, therefore, can stimulate initiatives to convert such industrial waste into value-added products.

## 5. Future Perspectives

This work supports the view that the effectiveness of drilling fluids containing different type of additives is significantly increased. When the conventional formulations for welding waste-derived additive-based formulations were substituted and the optimum concentration of additives was added, FL and MCT were reduced. The results prove that the novel welding waste-derived GO-based drilling fluids are capable of increasing efficiency, while reducing operating costs. This presents a golden opportunity to extract graphene from other industrial wastes and utilize it to further enhance drilling fluid properties. Welding wastes consist of toxic substances such as aluminum, titanium oxide and others. Metal oxides are hazardous to the environment and marine life. Hence, reuse and extraction into valuable products from raw welding waste is very important. In conclusion, lower amounts of toxins will be released into the environment, leading to a lower impact on the atmosphere.

Nanotechnology recently introduced new formulations of drilling fluids to the oil and gas industry. This technology can rearrange the properties of nanomaterials to produce more attractive properties which are essential in drilling fluids. By-products from the construction industry such as welding wastes can be reused in WBDFs and can serve as low-cost additives compared with commercial products in terms of optimizing the performance of conventional drilling fluids. The development of advanced methods is, therefore, required for the production of nanomaterials on a large scale and with cost-effective strategies for commercialization. In addition, proper guidelines for the handling and storage of nanomaterials must be implemented. This is to prevent contamination of the materials which leads to high surface activity and degrades their effectiveness.

Finally, novel routes are required to be discovered for the extraction of GO from waste materials. The use of welding waste-derived GO improves the rheological properties of drilling fluids. Researchers from different fields of expertise should use their skills and experience to improve technology and create new remarkable materials such as green nanocomposites. Other parameters such as high temperature and high pressure can also be used in future studies to investigate the effect of aging on the drilling fluids after the addition of novel additives.

## Figures and Tables

**Table 1:** Formulations of WBDF.

Materials	Basic WBDF	Basic WBDF + Waste Additives	Mixing Time	Mixing Order
Distilled water (mL)	300.0	300.0	-	1
Bentonite (ppb) [Al_2_O_3_.4(SiO_2_).H_2_O]	25.0	25.0	5	2
Potassium chloride (ppb)	20.0	20.0	2	3
Xanthan gum (ppb)	1.0	1.0	5	4
Polyanionic cellulose (ppb)	3.0	3.0	5	5
Sodium hydroxide (ppb)	0.1	0.1	5	6
Barite (ppb)	70.0	70.0	20	7
Welding waste (ppb)/ Welding waste-derived GO (ppb)/ Commercial GO (ppb)	-	0.01, 0.1, 0.5	5	8

**Table 2:** Rheological properties of commercial GO.

Concentration	Base WBDF	0.01 g	0.1 g	0.5 g
PV (cP)	14.0	16.0	16.5	17.0
AV (cP)	19.0	21.5	22.2	23.5
YP ($\mathrm{Ib}/100{\;\mathrm{ft}}^{2}$)	15.0	17.5	17.4	18.0
10sGS ($\mathrm{Ib}/100{\;\mathrm{ft}}^{2}$)	8.0	8.5	10.0	11.2
10mGS ($\mathrm{Ib}/100{\;\mathrm{ft}}^{2}$)	11.0	11.4	12.0	12.5
FL (mL)	9.0	7.4	7.0	6.8
MCT (mm)	2.82	1.52	1.44	1.13

**Table 3:** Rheological properties of raw welding waste.

Concentration	Base WBDF	0.01 g	0.1 g	0.5 g
PV (cP)	14.0	25.0	28.2	30.5
AV (cP)	19.0	28.0	30.4	31.4
YP ($\mathrm{Ib}/100{\;\mathrm{ft}}^{2}$)	15.0	15.0	16.2	17.2
10sGS ($\mathrm{Ib}/100{\;\mathrm{ft}}^{2}$)	8.0	7.0	8.0	8.5
10mGS ($\mathrm{Ib}/100{\;\mathrm{ft}}^{2}$)	11	9	10	10
FL (mL)	9.0	8.0	7.2	6.0
MCT (mm)	2.82	2.37	2.25	1.70

**Table 4:** Rheological properties of welding waste-derived GO.

Concentration	Base WBDF	0.01	0.1	0.5
PV (cP)	14.0	22.0	24.2	25.5
AV (cP)	19.0	24.0	26.4	27.0
YP ($\mathrm{Ib}/100{\;\mathrm{ft}}^{2}$)	15.0	17.0	18.2	19.0
10sGS ($\mathrm{Ib}/100{\;\mathrm{ft}}^{2}$)	8.0	6.0	6.5	7.5
10mGS ($\mathrm{Ib}/100{\;\mathrm{ft}}^{2}$)	11.0	8.0	9.5	9.7
FL (mL)	9.0	4.0	5.8	6.6
MCT (mm)	2.82	1.90	2.03	2.23

## Data Availability

Not Applicable.

## Abbreviations

API	American Petroleum Institute
AV	Apparent viscosity
EDX	Energy Dispersive X-ray analysis
PV	Plastic viscosity
YP	Yield point
GS	Gel strength
FL	Filtrate loss
FTIR	Fourier Transform Infrared Spectroscopy
FESEM	Field Emission Scanning Electron Microscopy
MCT	Mud cake thickness
GO	Graphene oxide
WBDF	Water-based drilling fluids
XRD	X-ray Diffraction Crystallography
